# Professor Berrutti on the Spontaneous Generation and Nature of the Spermatic Animalcules

**Published:** 1845-08

**Authors:** 


*Professor Berrutti on the Spontaneous Generation and Nature of the Spermatic Animalcules.*—The reproduction of certain animal and vegetable species is effected, without the intervention of any ovum, after the manner of what is called gemmiparous and fissiparous generation. Now there is a great analogy between the development of *gemmæ* and that of *ova*. In the former, however, all the elements, that are necessary to the production of a living being, are found contained; whereas, the latter have need of the new elements of the prolific fluid before they can become duly evolved.

Every particle of a living being—which, on being detached from its parent body, is capable of reproducing an independent living creature—does not materially differ from a bud, either in its mode of origin, or in its property of producing new individuals. Thus organic molecules, in certain circumstances, have the power of attracting to themselves new materials from surrounding bodies, which they then incorporate with themselves, and so elaborate as to form a new being. Spontaneous generation, in the opinion of Professor Berrutti, consists in the exercise of this property, and differs from oviparous, gemmiparous and fissiparous generation, in that it takes place among molecules which, in consequence of the death of the parent, have ceased to constitute a part of a living individual.

Microscopic observations have clearly shewn that the globules, resulting from the dissolution of organic matter, possess an inherent activity: sometimes they approach to and unite with each other, and at other times they seem to be mutually repellent. It is not at all inconsistent with rational belief to suppose that this is the cause of spontaneous generation—an act, it may be observed, to which the concurrence of the atmospheric air and of water is probably always necessary.

The reproduction of parts of an organic body that have been excised or destroyed is effected by means of globules floating in a fluid, which subsequently evaporates, leaving the globules dry: hence the cellular production and origin of new tissues. The same process of assimilation is likewise that by means of which the fœtus is formed in the maternal ovum, as Wolf and Rolando have shewn.

The necessary condition, therefore, of every sort of generation, reproduction, and even of the nutrition of organised parts is invariably the presence of certain organic globules endowed with a plastic activity, and floating in a fluid exposed to the contact of the atmospheric air—which, in place of furnishing carbonic acid as it does to plants, supplies ammonia for the spontaneous generation of animals. The fluid in its turn supplies hydrogen and oxygen.

The organic productions of spontaneous generation are the most simple of all, because they spring from organic globules that are not expressly prepared for this purpose.

Professor Berrutti is of opinion that not only infusory, but also entozoary, animalcules are developed by spontaneous generation. He considers it too as highly probable that the *acarus scabiei* is the product, rather than the cause, of the itch. Lastly, he seeks to shew that the Zoosperms are not genuine animalcules, but rather organic molecules formed in the minute extremities of the spermatic tubes by the effect of an exuberant nutrition. The action of the Zoosperms seems to be very analogous to that of the Pollen in the fecundation of plants; and their movements may fairly be compared to those of this vegetable matter.—*Ibid*, *from Annali Univ. di Medicini.*

